# Medical International Congress

**Published:** 1866-09

**Authors:** M. Bouillard, M. Jaccond

**Affiliations:** President; Paris; Secretary; Paris


### MEDICAL INTERNATIONAL CONGRESS.

A Medical Congress, to meet in Paris at the World's Fair in April next, is proposed. A communication has been received, addressed to us by the Committee appointed to communicate with medical and other scientific organizations in other nations. We invite attention to the subject, and would be pleased to publish any suggestions from the Profession on the subject.

The following is the translation of the communication referred to:

PARIS, June 1st, 1866.

*Messrs. Editors:* A Medical International Congress will convene at Paris, in 1867, at the meeting of the World's Fair The time has now arrived for making known to our confreres, both the project itself and that which has already been done to insure its success. Under these circumstances, we think it advisable to give it publicity through your estimable Journal, and we now ask that you have the kindness to insert the note which we have the honor to address you. In November last, a Central Committee was formed at Paris, for the purpose of preparing an organization for the Congress of 1867, and replying to the petitions sent by the Congress of Bordeaux. The members of the Committee are:

M.M. E. Borthez, Beclard, Behier, Bonchordat, Bouillard, Broca, Dechambre, Denonvilliers, Follin, Gavonet, Gosselin, Jaccond, Lasegne, Longet, C. Robin, Cordien, Verneuil, E. Vidal, and Wurtz.

The Bureau was permanently organized December 7th, by the appointment of the following officers:

M. Bouillard, President;

M.M. Denonvilliers, Gavonet, and Cordien, Vice-Presidents;

M. Jaccond, Recording Secretary;

M. E. Vidal, Corresponding Secretary.

The organization being completed, we requested the Minister of the Interior for authority to carry out the designed project; this authority was granted us March 20th. The Committee of the Bureau immediately reported to the Minister of Public Instruction, who not only gave his entire approbation to this scientific work, but his eminent patronage. The Minister of Agriculture and of Commerce received with no less favor the communication we had the honor to make him; finally, the Minister of Foreign Affairs has given us his support, and promised to recommend the Congress to the representatives of France abroad. Such, Messrs. Editors, is the state of affairs, and we are assured that these eminently favorable conditions are of themselves powerful guarantees of success. But, on the other hand, the Congress derives from its special character, an importance which cannot be misunderstood.

Passing the limits of nationality, between which have been closed, until now, medical assemblies, the National Congress of Paris will not be a simple convention of Doctors, but it will be the consummation of the scientific movement of our age, and the first visible act of that intellectual alliance which will unite the laborers of every country.

Knowing the devotion and the zeal of the medical press to the true interests of science, we therefore presume that its valued concurrence will not be withheld in the important affair. In its approaching meeting, the Committee will be employed in the elaboration of the regulations and of the programme of the Congress, which, when completed, we will have the honor of communicating to you. Accept, Messrs. Editors, our thanks and assurances of our highest regard.

In behalf of the Committee:

M. Bouillard, *President.*

M. Jaccond, *Secretary.*

